# Time‐varying nodal measures with temporal community structure: A cautionary note to avoid misinterpretation

**DOI:** 10.1002/hbm.24950

**Published:** 2020-02-14

**Authors:** William Hedley Thompson, Granit Kastrati, Karolina Finc, Jessey Wright, James M. Shine, Russell A. Poldrack

**Affiliations:** ^1^ Department of Psychology Stanford University Stanford California; ^2^ Department of Clinical Neuroscience Karolinska Institutet Solna Sweden; ^3^ Centre for Modern Interdisciplinary Technologies Nicolaus Copernicus University in Toruń Toruń Poland; ^4^ Department of Philosophy Stanford University Stanford California; ^5^ Brain and Mind Centre The University of Sydney Sydney New South Wales Australia

**Keywords:** integration, network neuroscience, participation coefficient, temporal network, time‐varying connectivity

## Abstract

In network neuroscience, temporal network models have gained popularity. In these models, network properties have been related to cognition and behavior. Here, we demonstrate that calculating nodal properties that are dependent on temporal community structure (such as the participation coefficient [PC]) in time‐varying contexts can potentially lead to misleading results. Specifically, with regards to the participation coefficient, increases in integration can be inferred when the opposite is occurring. Further, we present a temporal extension to the PC measure (temporal PC) that circumnavigates this problem by jointly considering all community partitions assigned to a node through time. The proposed method allows us to track a node's integration through time while adjusting for the possible changes in the community structure of the overall network.

## 1 INTRODUCTION

Quantifying the time‐varying properties of a network often utilizes a multilayer network approach of temporally ordered “snapshots” consisting of connectivity matrices through time (i.e., temporal network theory; Holme & Saramäki, 2012; Kivelä et al., 2014). This approach answers questions about how nodes, edges, and communities in a network fluctuate over time. In recent years, such temporal network approaches have increased in neuroimaging yielding new insights about the brain (Shine & Poldrack, 2018). Importantly, to generate knowledge about an underlying empirical network, the temporal network measures must be mapped back to, or interpreted in terms of, the phenomenon they are modeling.

There are many metrics available for quantifying topological features of nodes within temporal networks. Some measures are temporal extensions of static measures (e.g., *TempoRank* is a temporal extension of *PageRank* [Rocha & Masuda, 2014]). Others apply static measures to each temporal snapshot (e.g., Bola & Sabel, 2015 found changes in rich club coefficients applied to multiple time points). In this latter case, it is essential to ensure that the interpretability or clarity of the measure is not changed or distorted when used through time.

**Figure 1:**
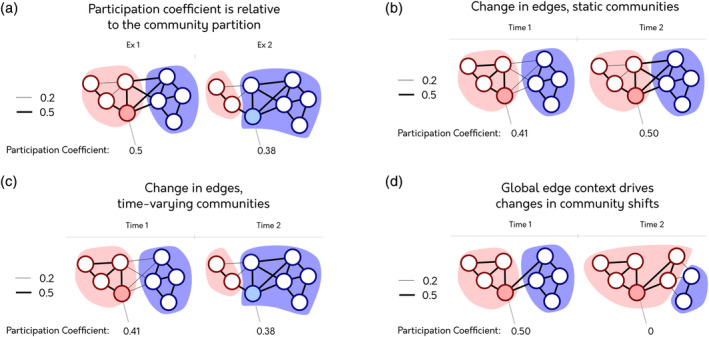
Different ways to calculate the participation coefficient (PC) through time. The PC for the shaded node is below each network/temporal snapshot. The border of each node and the colored backgrounds show the assigned community of each node. In this example, there are two different possible edge weights. (a) Two examples of the PC, illustrating how the measure is calculated relative to the community partition. (b) An example of PC calculated when applying a static community template across multiple temporal snapshots (*PC*_*S*_). (c) An example of PC calculated when applying a temporal community partition to multiple temporal snapshots (*PC*_*T*_). PC values between time points cannot be directly compared due to differences in community structure obtained for each time point. (d) An example showing that changes in community partitions over snapshots may result from changes in edges that do not directly connect to the node of interest. The difference in community partition is the result of changes in the nodes in the blue community. These changes affect the PC of the shaded node despite the fact that there was no change in its connectivity

The participation coefficient (PC) is an example of a static network measure used in time‐varying contexts that is applied to multiple temporal snapshots. Briefly, the PC quantifies the diversity of a node's connections to other nodes across a community partition (Guimerà & Nunes Amaral, 2005). The community partition groups different nodes based on a grouping property (e.g., modularity, when tightly connected nodes form modules [Newman & Girvan, 2004]). Importantly, the PC for any given node is relative to the community partition used to calculate it (Figure 1a); if the community partition changes, then the PC may also change. In the two examples in Figure 1a, the shaded node has the same edges, but the communities are different, entailing that the PC changes.

Community partitions can also be calculated through time. Temporal communities can merge, split, disappear, and reappear through time (Granell, Darst, Arenas, Fortunato, & Sergio, 2015). In the brain, the community structure has been shown to change in response to task and cognitive demands (e.g., Braun et al., 2015; Thompson, Wright, Shine, & Russell, 2019; Vatansever, Menon, Manktelow, Sahakian, & Stamatakis, 2015).

Considering the above, it is understandable that many previous studies have applied the PC through time while using temporal communities (e.g., Betzel, He, Rumschlag, & Sporns, 2015; Fukushima, Betzel, He, & van den Heuvel, 2018; Fukushima & Sporns, 2018; Pedersen, Omidvarnia, Jackson, Zalesky, & Walz, 2017; Rizkallah et al., 2019; Shine et al., 2016; Shine, van den Brink, Hernaus, Nieuwenhuis, & Poldrack, 2018; Tanimizu et al., 2017; Xie et al., 2018). However, a problem with the interpretation of the PC emerges when comparing two (or more) snapshots of a network with different community partitions. When community boundaries are allowed to fluctuate, the PC estimates through time are calculated relative to different community contexts. Here we argue that calculating PC per time point with a temporal community structure does not necessarily quantify its intended property of integration. As a result, the crucial link between the quantified measure and its mapping to the empirical phenomenon breaks down. Consequently, definitive conclusions cannot be drawn about what is happening in the brain.

We demonstrate this problem on toy network examples and then using resting‐state fMRI data. Finally, we propose a new method—the temporal PC (TPC)—that takes into account various community partitions calculated over time.

## 2 METHODS

### 2.1 Data used and data accessibility

We used data from the Midnight Scan Club resting‐state fMRI (Gordon et al. 2017) that is publicly available onopenneuro.org (ds000224). The data is available after both preprocessing and denoising steps have been performed (see Gordon et al. 2017 for details of these steps). The data consists of ten subjects that underwent ten resting‐state fMRI runs. One subject was excluded due to substantial artefacts. We extracted time series from 200 functionally‐defined parcels (Schaefer et al.2018).

### 2.2 Time‐varying connectivity estimation

We estimated time‐varying connectivity using weighted Pearson correlations for each time point. Weights were determined by using the Euclidean distance between all other time points. Briefly, the method creates a T × T matrix containing the distance between all nodes at each time point. At *t*, the corresponding row of the distance matrix becomes a weight vector for the connectivity estimates at *t*. The weight vector (W) is then are first converted into a similarity matrix (1‐W) and then scaled between 0 and 1. This vector is then used as the weights in the covariance matrix to create a weighted Pearson estimate of each edge at each time point (see Thompson, Brantefors, & Fransson, 2017 for more details). Comparatively, the sliding window approach uses “temporally nearby” time points to support its connectivity estimates when estimating the covariance, this method uses “spatially nearby” time points to support its connectivity estimate (see Thompson & Fransson, 2018 for illustrations). We selected this method of functional connectivity estimation because it performs well (second place) at tracking a fluctuating covariance through time in simulations (Thompson, Richter, Plavén‐sigray, & Fransson, 2018). The method that was ranked first—the jackknife correlation—was not chosen here because it calculates a time series of “differences in connectivity” which has a different interpretation than most other methods and deemed inappropriate for the method‐independent conclusions that we intend to make.

Prior to calculating the Louvain community detection and the PC, all edges below 0 were set to 0. While there are ways to calculate the PC and communities with negative edges, this is a common step that many of the cited studies we are addressing do.

### 2.3 Quantifying community structure

We calculated the temporal communities using the Louvain algorithm (Blondel, Guillaume, Lambiotte, & Lefebvre, 2008) with a resolution parameter of 1. Temporal consensus clustering was performed by assigning communities at time *t*‐1 with the same label as the community at time *t* that had the smallest Jaccard distance (Lancichinetti & Fortunato, 2012). We also calculated static functional connectivity using Pearson correlations and a static community partition with the same parameters as the temporal communities.

### 2.4 Static PC

The PC is defined by Guimerà & Nunes Amaral, (2005) as:

$$P_{i}=1-\sum_{s}^{N_{m}}{\left(\frac{k_{\text{is}}}{k_{i}}\right)}^{2}$$

where *i* is a node index and *N*_*M*_ is the number of communities. *k*_*is*_ is the within‐community degree, and *k*_*i*_ is the overall degree of node *i*.

### 2.5 The PC through time with static communities (PC_s_)

When calculating the PC through time, with static communities, the equation is:

$$P_{\mathrm{it}}=1-\sum_{s}^{N_{m}}{\left(\frac{k_{\mathrm{its}}}{k_{\mathrm{it}}}\right)}^{2}$$

Here, we see temporal subscripts for the participation and the degree of the nodes. Note that, there is only one community partition used for all time points.

### 2.6 The PC through time with temporal communities (PC_T_)

The PC with temporal communities is:

$$P_{\mathrm{it}}=1-\sum_{s}^{N_{\mathrm{mt}}}{\left(\frac{k_{\mathrm{its}}}{k_{\mathrm{it}}}\right)}^{2}$$

Above, we are summing over the communities *N*_*mt*_ which is the number of communities found at time point *t*. This method uses a community partition calculated separately per time point.

### 2.7 Temporal PC

The TPC that we introduce is:

$$P_{\mathrm{it}}=1-\frac{1}{T}\sum_{u}^{T}\sum_{s}^{N_{\mathrm{mu}}}{\left(\frac{k_{\mathrm{its}}}{k_{\mathrm{it}}}\right)}^{2}$$

Here, we have added that all temporal community partitions are considered for each time point. See the results section for the motivation behind the TPC.

We abbreviate the different PC methods as follows: “the static participation coefficient” (static *PC*), “the participation coefficient per time point with static communities” (*PC*_*S*_), “the participation coefficient per time point with temporal communities” (*PC*_*T*_), and “the temporal participation coefficient” (*TPC*).

### 2.8 Additional network measures

We also related the different participation measures to different network estimates throughout the article to help interpret what the changes in participation means for the network. The additional measures we used were: (a) flexibility (the percentage of times where a node changes its community, Bassett et al., 2011); and (b) within‐module degree z‐score (*z*; z‐score of node's degree within its community, Guimerà & Nunes Amaral, 2005). We calculated *z* in two ways: using static communities (*z*_S_) and using temporal communities (*z*_T_). We combined the different *z* estimates with their methodological participation counterpart (i.e., *z*_*S*_ is used with *P*_*s*_).

### 2.9 Illustrating the methodological choices do not impact the conclusion

To ensure that our concerns about *PC*_*T*_ generalize across different methods, we replicated part of the analysis (Figure 5a) by changing some methodological choices: (a) the time‐varying connectivity estimation method, and (b) the community detection algorithm. As an alternative time‐varying connectivity estimation method, we used the multiplication of the temporal derivative (MTD) method (Shine et al., 2015). This method multiplies the temporal derivatives of each pair of time series and applies a smoothing parameter that averages over ±4 time points. When changing the community detection algorithm, we applied the “temporal community by trajectory clustering” method (TCTC) (Thompson et al., 2019, with parameters: *ɛ*: 0.5, *σ*: 5, *τ*: 5, *κ*: 1). Since this community detection is multilabel, we forced a single label partition by applying the Louvain algorithm (resolution parameter: 1) to drive each node into a single community.

## 3 RESULTS

### 3.1 Different reasons underlying changes in PC through time

Here, we illustrate the potential problem introduced when calculating the PC through time on toy network examples. Consider a time series of PCs when the community partition is static (Figure 1b). For the two different temporal snapshots, there is a change in the edges of the shaded node, which changes the PC of that node. Specifically, in the second snapshot, the connections of this node have become evenly distributed across all communities. We can easily relate the two PC values for the two different snapshots to each other, and it makes sense to interpret the increase PC as an increase in the node's interaction with communities outside of its own.

If instead, the community partition varies over time (Figure 1c), the changes in edges lead to the shaded node being classed as part of the blue community instead of the red community. The node's PC, in light of this change in community membership, is reduced. This decrease happens because the node changes community membership when it increased its connection strength with the blue community. Hence, the interpretation of a time series of PCs as reflective of a change in intracommunity connections is impeded by the extent the community structure is changing over time. The problem is that the *PC*_*T*_ values in a time‐series can no longer be directly compared without additional information to understand how the abstract measure maps back to the external phenomenon.

A possible objection to this criticism of *PC*_*T*_ is that the temporal communities are calculated on the edges themselves, entailing an interconnection between the community context and edge context of a node. This objection does not adequately take into account how communities are generally calculated. Communities take into account the “global edge context” (i.e., all edges in a network and how they relate to each other) whereas the PC only considers the “local edge context” (i.e., all edges connected to one node). There is no necessary relationship between these two (exemplified in Figure 1d). A node's strength can increase with no effect on the community partition. Alternatively, a node can change its community assignment with no change to its own edges.

### 3.2 Misleading network‐level interpretations of PC with temporal communities

We have demonstrated that community context affects a node's PC when quantified at multiple snapshots. Here, we show how *PC*_*T*_ can cause misinterpretations. To address this, we must first consider what property that PC tries to identify. In its introduction by Guimerà and Nunes Amaral (2005), they claim that “[t]he participation coefficient *P*_*i*_ measures how ‘well‐distributed’ the links of node *i* are among different modules” (p. 897). This property of “well‐distributed” edges has been interpreted as the *integration* within network neuroscience when applied to static functional connectivity. For example, Bertolero et al. (2017) interpreted their results regarding *PC* as: “nodes with high PCs integrate information and coordinate connectivity between communities” (p. 2). Likewise, Power et al. (2013) stated that the PC's interpretation relates to information spanning multiple different systems: “If a node has a high participation index […] we infer that such nodes likely have access to a variety of types of different information processing represented among different systems” (p. 808). *PC*_*T*_ kept the same interpretation when applied in time‐varying contexts. Shine et al. (2016) stated that they identified “functional states that maximize either segregation into tight‐knit communities or integration across otherwise disparate neural regions” (p. 544) where the integration was calculated using *PC*_*T*_. Thus, from its network science origins, to static functional connectivity in network neuroscience, to time‐varying connectivity, the PC has consistently been used to quantify the integration of nodes in a network.

**Figure 2:**
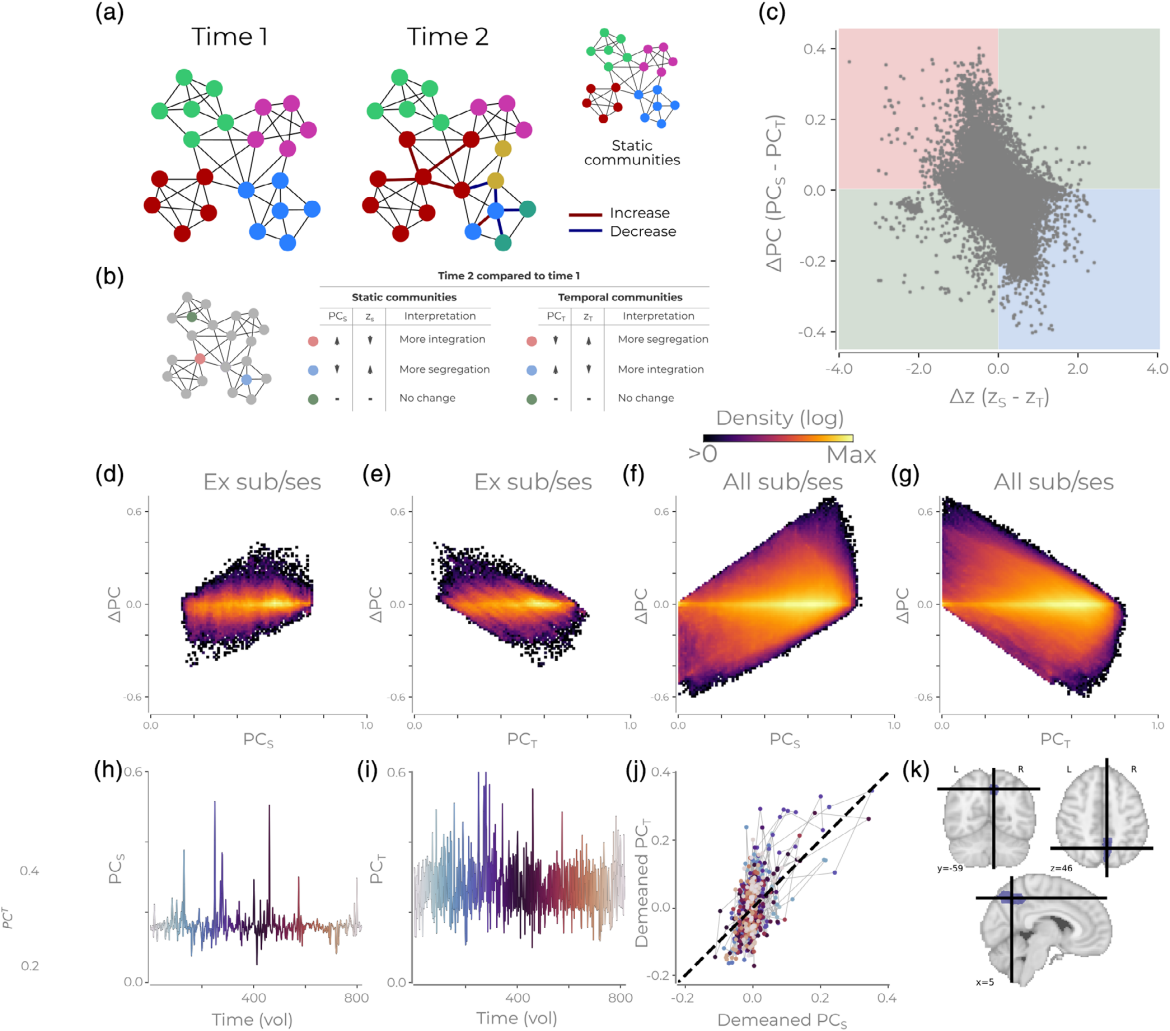
The different PC calculation methods lead to different interpretations of the network's organization. (a) An example network consisting of multiple communities for two time points. The figure shows both a temporal community partition and a static community partition. Red lines at time point 2 indicate an increase in connectivity, and blue lines indicate a decrease. (b) Different types of nodes from (a) are highlighted. The accompanying table states how these nodes at time point 2 will be quantified relative to time point 1 with static or temporal communities for the participation coefficient (PC) and within‐module degree z‐score (z). (c) The difference in PC and z for nodes in the static and temporal for all nodes/time points for one subject/run in the MSC dataset. The red, blue, and green quadrant corresponds to the interpretations found in (b). (d) Density plot showing the difference in PC versus *PC*_*S*_ for the example session/subject in (c). (e) Same as (d) but the difference in PC versus *PC*_*T*_. (f) Same as (d) but for all sessions/subject. (g) Same as (e) but for all sessions/subjects. All density plots have a logarithmic color scale. (h) The *PC*_*S*_ through time for an example subject/session/node. The color of the time series is to assist understanding of Panel (k) and changes as the time series progresses. (i) Same as (h) but for the *PC*_*T*_. (k) The *PC*_*S*_ and *PCT* from panels (h) and (i) plotted against each other. The color of each point corresponds to the time illustrated marked in (i,h). (l) Depiction of the example node used in Panels (h–k)

We will now show that multiple estimates of *PC*_*T*_ do not identify moments of increased integration for a node. Consider the toy network shown in Figure 2a. Here we have two different time points where the community context changes. In this toy example we have, for simplicity, only marked edges that increase or decrease at the second time point relative to the first. We have selected nodes from this network and identified if, with *PC*_*S*_ and *PC*_*T*_, they exhibit increased integration, segregation or no change in regards to the previous time point (Figure 2b). Note how *PC*_*T*_ assigns increased participation, and thus interpreted as having higher integration, to the node marked in blue in Figure 2b. The difference between the network at Time 1 and Time 2 is the blue community which has split into two smaller communities. Following the interpretation that integration represents the information processing across different communities (e.g., quote by Power et al. 2013), it is hard to consider a split in community structure due to a decrease in the magnitude of edges as increased integration. Likewise, the temporal community of the red node has extended in the second time point (Figure 2b, Time 2). This extension of the community can be interpreted as the red node sharing similar information with more nodes due to the increase in edge weights (i.e., integration). However, *PC*_*T*_ will ascribe a low score and interpret less integration at the second time point. In both these examples, *PC*_*S*_ provides the opposite interpretation to *PC*_*T*_. Given the definitions of integration, we see that moments of increased *PC*_*T*_ cannot be interpreted as moments of more integration in the brain.

Note that, there is nothing mathematically wrong with each *PC*_*T*_ estimate in isolation—each estimate is indeed correct. The problem lies when contrasting *PC*_*T*_ estimates with each other and inferring changes in integration across time. When quantifying a time series of participation estimates, it is typical for such between time point inferences to be made.

Do situations where *PC*_*S*_ and *PC*_*T*_ have flipped interpretations occur on empirical data (i.e., situations demonstrated in Figure 2b)? To test this, we calculated the difference between *PC*_*T*_ and *PC*_*S*_ and between *z*_*T*_ and *z*_*S*_ on an example subject's resting‐state fMRI data (Figure 2c). In the figure, the colored quadrants represent situations similar to Figure 2b's node examples. Here, the red quadrant indicates that they would have “more integration with static communities, more segregation with temporal communities” and vice versa for the blue quadrant. A large portion of the nodes (example session/subject: 75.59%; all subjects: 66.08%) end up in the red and blue quadrants, entailing that they have alternative interpretations about what is occurring in the network when using *PC*_*T*_ and *PC*_*S*_ methods.

A possible critique of the foregoing analysis is that the differences we highlight reflect a well‐known negative relationship between PC and *z* and we are merely illustrating small deviations between the two methods while preserving the static relationship. However, our argument is that different time points receive substantially different interpretations between the two PC methods, and not to draw any conclusion about the overall negative relationship between the differences of PC and *z*. To clarify this, we found that when difference in PC is large, then either *PC*_*T*_ or *PC*_*S*_ will have high participation (Figure 2d–g). Thus, we see that the two PC methods are interpreting time points differently which is all we are trying to establish in this section. For completeness, we further show the PC trajectories through time for a single node (Figure 2h–k). The time series for PC_S_ (Figure 2h) and *PC*_*T*_ (Figure 2i) are *ostensibly* different. Contrasting the two (demeaned) example time series highlights that *PC*_*T*_ has multiple peaks of participation when *PC*_*S*_ interprets the time point as average or below in participation (Figure 2j).

In sum, we have shown that there is a divergence between the methods. This divergence entails that *PC*_*S*_ and *PC*_*T*_ give different interpretations about a node's role in the network through time. Further, we have shown that differences in *PC*_*T*_ across time do not map to increases in integration. Thus, given the PC's long history with being used to quantify the amount of integration in the brain, using *PC*_*T*_ will lead to misinterpreting results. Importantly, *PC*_*S*_ does not suffer from this problem as it uses the same reference community. However, the temporal community information is discarded, leading us to our possible modification of PC to allow for temporal communities.

### 3.3 PC in relation to all temporal communities

**Figure 3:**
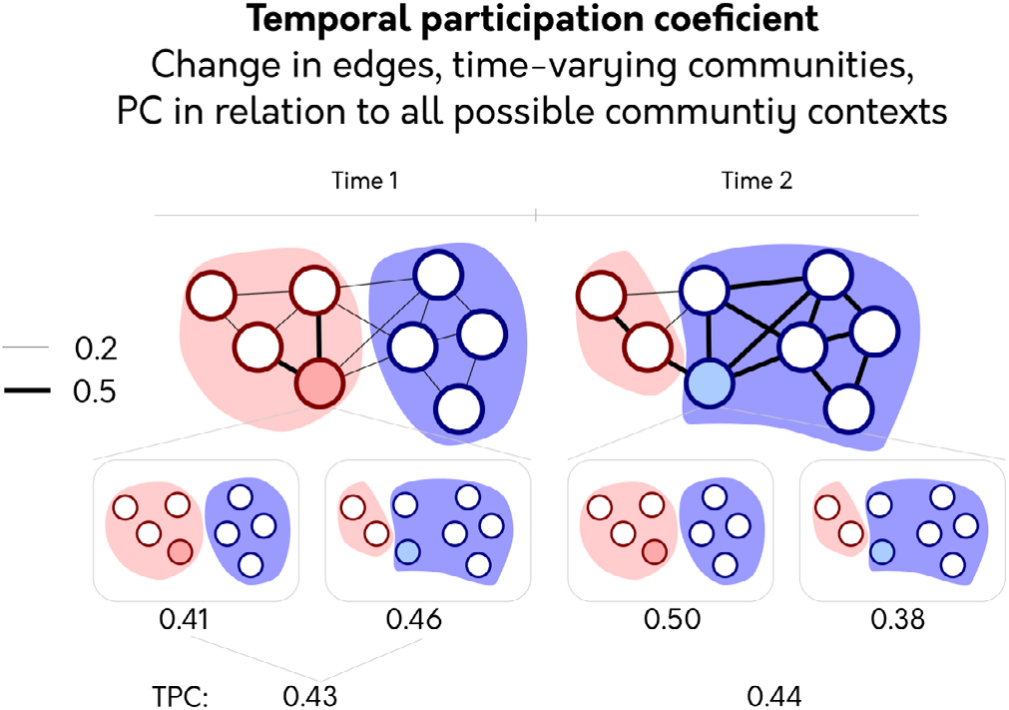
The temporal participation coefficient (*TPC*). To calculate the *TPC*, PC is calculated per time point, taking into account all possible community contexts the node can be in. PC value calculated for each possible temporal community context is shown under each network. These are then averaged together. This fix makes PC of the shaded node comparable across time points as they have been compared against the same community contexts

Given the substantial evidence for temporal changes in community structure, there is an understandable desire to calculate the PC with fluctuating communities. We present a possible solution to the problem outlined above: the TPC. The crux of the problem is that the PC of a node is relative to the community partition. If instead, each PC estimate considers all possible community partitions that the node has been assigned, then the multiple PC estimate will be comparable across time points as each estimate is now relative to the same community context (Figure 3). Specifically, *TPC* considers how a node is participating relative to all possible community structure it can have. In Figure 3, each *TPC* estimate is calculated relative to both community contexts and then averaged, entailing that the shaded node at the second time point has more participation compared to the first time point (contrast to *PC*_*T*_ in Figure 1). As both time points are considered relative to both community contexts, these values can now be compared.

The motivation behind *TPC* is to have all time points compared to the same community context (like *PC*_*S*_) but to retain the temporal information inherent in community fluctuations (used in *PC*_*T*_). Perhaps this logic seems counter‐intuitive as it entails using community partitions that do not fully reflect the snapshot. However, this is also the same logic as *PC*_*S*_, as it applies a static community partition to each time point that does not fully reflect the community structure at each snapshot. The only difference here is that *TPC* utilizes multiple community partitions instead of a single partition.

*TPC* quantifies a node's activity in relation to all communities that it could potentially be in. Consequently, if a single large edge at *t*_*1*_ merges two communities and, at *t*_2_, only that edge decreases such that the community splits, *TPC* will always assign higher participation to *t*_1_. Since all time points use the same community information, it is mathematically impossible for any time point that decreases all of its edge weights to get higher participation—this guarantee is not possible for *PC*_*T*_. Thus, *TPC* can utilize all community contexts but avoids the problematic applications shown for *PC*_*T*_ in the previous section.

There is one crucial assumption when applying this method, namely that the community structure can recur again through time. This assumption means that the same or similar community partitions will be found at later time points. In a network like the brain, this is reasonable, and it is a basic assumption that is made in all psychological experiments where the task is repeated (see discussion for when this assumption breaks down). Without this assumption, it becomes unfair to use earlier or later community partitions to analyze a node's PC in a snapshot as if it was in another community.

### 3.4 Nodes with high static PC change communities the most

We have presented a theoretical problem, and illustrated how it could lead to differences in interpretation. We have also proposed a fix that does not suffer from interpretation problems. However, it *could* be the case that there is no difference when applying *TPC* versus *PC*_*T*_.

**Figure 4:**
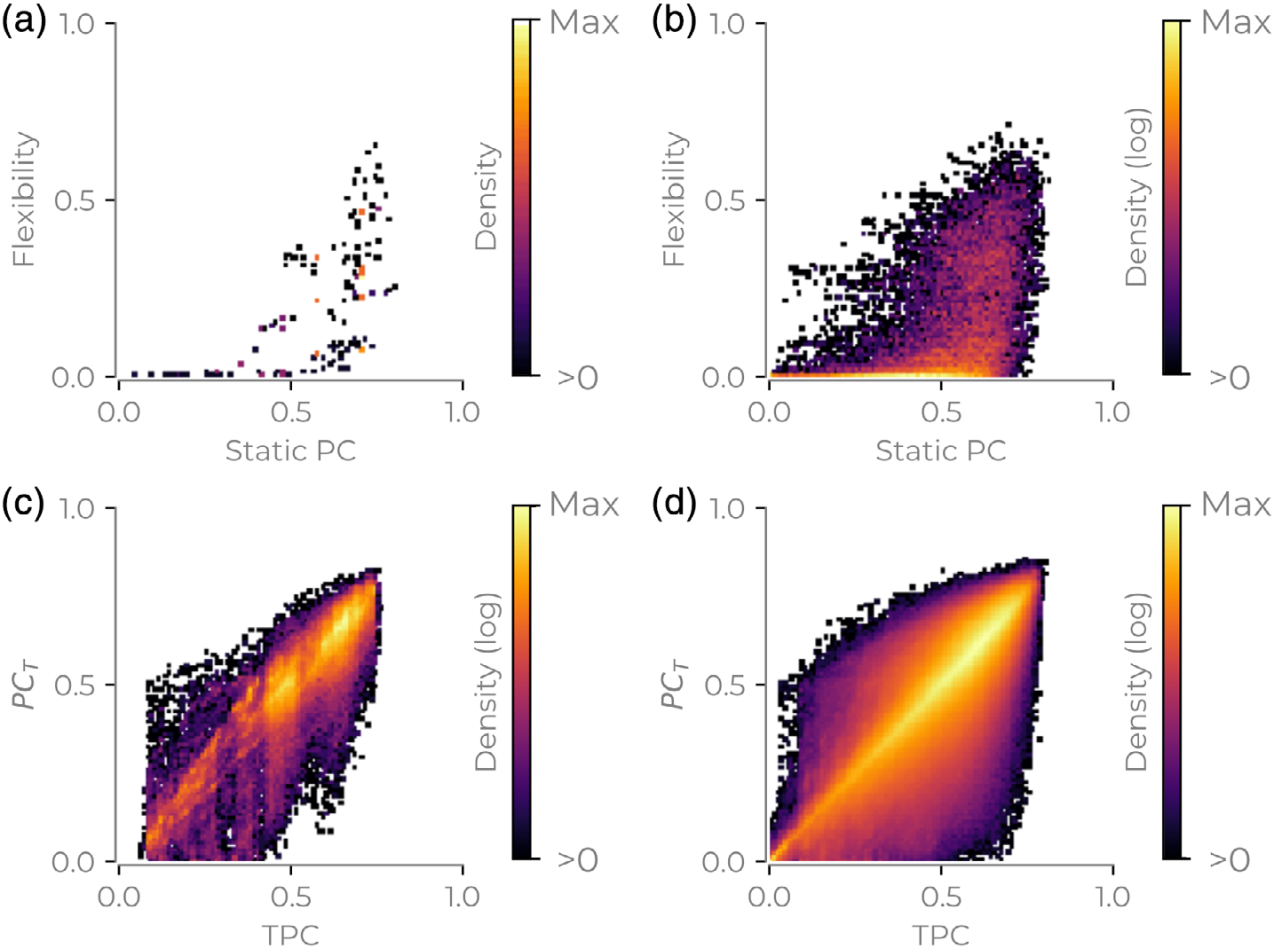
Differences between *TPC* and *PC*_*T*_. (a) Static participation coefficient (PC) versus the flexibility for one subject/session. (b) Same as (a) but for all subjects. (c) Temporal PC versus the PC per time point with temporal communities for one subject/session. (d) Same as (c), but for all subjects. All color bars show density on a 100 × 100 grid. Panels (b–d) show logarithmic range between colors

We begin by asking the question: how are nodes with high static PC affected by the temporal community partitions? If nodes with high participation have little change in their community context, the problem we raise may be redundant. To clarify this, we compared the flexibility with the static PC (Figure 4a,b). Here, we see that nodes with high participation also increase their flexibility. If nodes with high participation always remained in the same communities, calculating PC with temporal communities would be less problematic. Nodes that switch temporal communities have high static PC. We have not proven which participation method should be preferred here. However, this relationship that the changes in communities are affecting nodes with high participation which will help us understand any differences between participation methods.

### 3.5 Divergence of the different methods

Now, we contrast *PC*_*T*_ with the *TPC* to see whether they compute similar values or whether they diverge. First, we begin by considering all time points and all nodes together. A heteroscedastic relationship between the two coefficient emerges (Figure 4c,d, Bartlett test for heteroscedasticity: all subjects: *T* = 153,221.9, *p* < .001; example subject: *T* = 1902.3, *p* < .001). This heteroscedastic relationship entails that, while both methods may identify points that have the highest participation, the relationship quickly breaks down. For completeness, Supplementary Figure S1 shows the correlation between both *PC*_*T*_ and *TPC* with *PC*_*S*_. It can be observed that the extent of the heteroscedastic spread is largest for *PC*_*S*_ and *PC*_*T*_.

**Figure 5:**
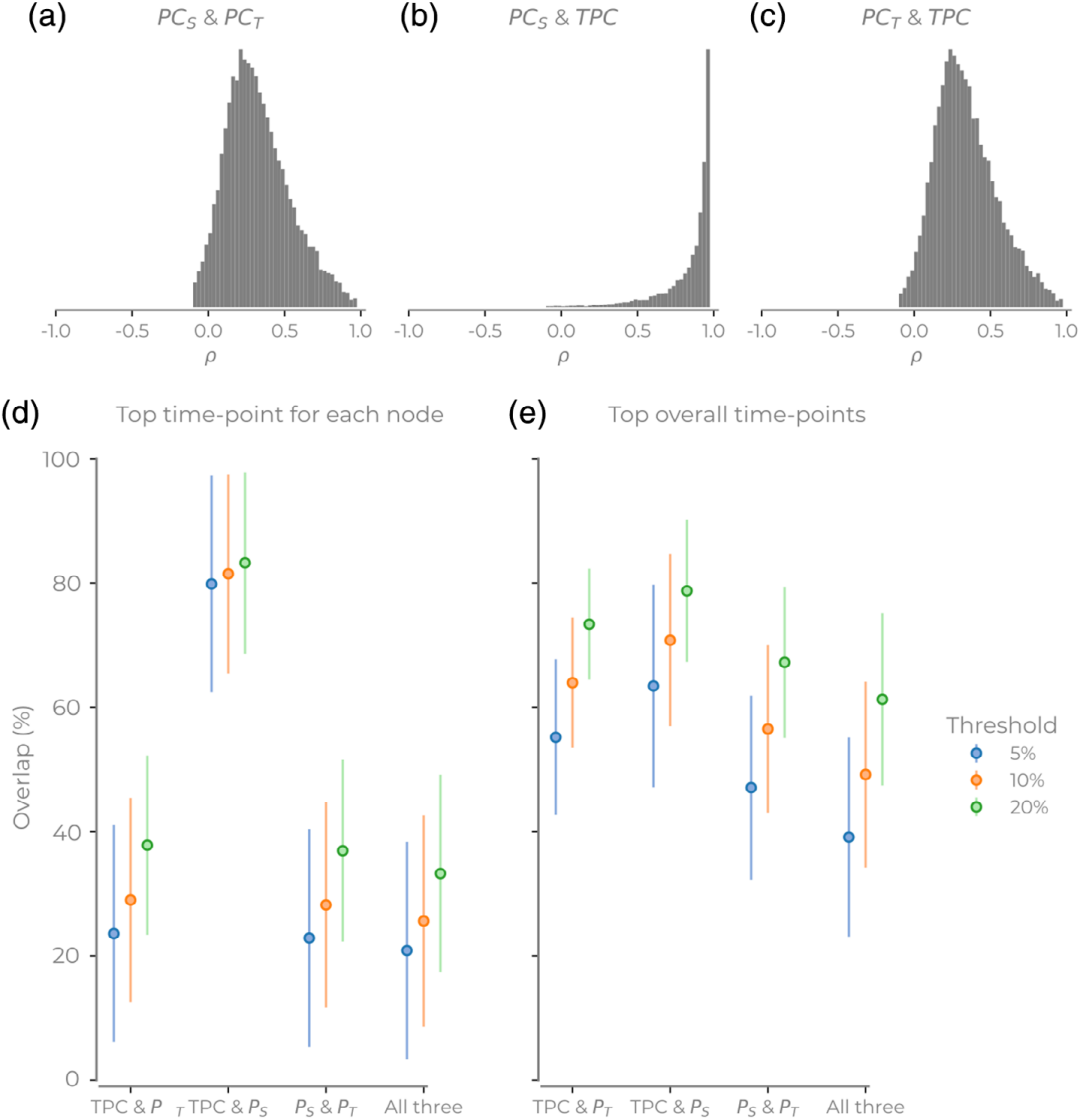
Comparison of the hub overlap for three different participation coefficient (PC) methods. (a–c) The histograms of correlation values for each time series for different participation methods. Histograms show all nodes, sessions, and subjects. (d,e) The intersection of high participation coefficient from different methods. Here, we see the intersection of each combination of three methods. (d) For each subject, the top *x*% time point for each node. (e) For each subject, the top *x*% across all nodes and time points. Error bars show *SD*

To quantify the extent to which the methods diverge, we considered two different questions: (a) do the time‐series of PCs correlate with each other?; and (b) if selecting the top *x*% of time points to be marked as candidate temporal hubs for the different methods, do the selections intersect? The time series of *PC*_*S*_ and *PC*_*T*_ did not correlate highly (Spearman rank [*ρ*]: median: 0.30, *SD*: 0.21, min: −0.44, max: 1.0, Figure 5a), especially compared to *PC*_*S*_ and *TPC* (Spearman rank: median: 0.96, *SD*: 0.19, min: −0.80, max: 1.0, Figure 5b). *TPC* also did not correlate highly with *PC*_*T*_ (median: 0.32, *SD*: 0.21, min: −0.37, max: 1.0, Figure 5c). Note that, the descriptive statistics in both *PC*_*T*_ and *PC*_*S*_ with *TPC*, the minimum values can be negative, but the number of these negative correlations is few in number (hence not visible in Figure 5b,c). In sum, these correlations show that *TPC* and *PC*_*S*_ correspond the most with each other through time. However, while there is generally a positive correlation between *TPC* and *PC*_*S*_ time series, some of the time series differ quite radically (i.e., min correlation value is −0.80). This illustrates that these two methods can produce very different results for some nodes.

Taking the average PC over time for the various methods, the correlation between the methods is high for all combinations (*PC*_*S*_ and *PC*_*T*_: 0.91; *PC*_*S*_ and *TPC*: 0.91; *PC*_*T*_ and *TPC*: 1.0). This shows that the average PC over time is similar across methods. Thus, the divergence of the methods is primarily with the fluctuations through time.

The correlation between PC time series does not mean that the same nodes or time points will be candidate hubs. For each method, we then identified the highest 5, 10, and 20% of values for both for the top time points for each node (Figure 5d) and when concatenating across all nodes (Figure 5e). If we try and find when each node has its highest participation, we find that *PC*_*T*_ has more unique nodes. When pooling all nodes and time points together, the overlap of all three methods reached over 60% with larger thresholds, but was under 40% for lower thresholds. Finally, we also observed that the *TPC* and *PC*_*S*_ overlapped the most (reaching 80% of nodes in some instances and always over 60% when combining the paired and triple intersections). This overlap is reassuring for *TPC* as we know *PC*_*S*_ is a valid method. Moreover, the divergence that happens between the *TPC* and *PC*_*S*_ with static communities is due to the *TPC* utilizing the temporal community information.

Finally, we replicated Figure 5a by changing the time‐varying connectivity method (to MTD) (Supplementary Figure S2a) and by changing the community detection algorithm (to TCTC) (Supplementary Figure S2b). The general pattern remains when contrasting Figure 5a with these other methods. While the distributions changed shape for *PC*_*S*_ and *TPC*, the peaks of the distributions remained in the same order as Figure 5a. More importantly, there is no sizeable improvement with *PC*_*T*_'s correlations with the other methods, entailing that the interpretation problem with *PC*_*T*_ persists. In sum, methodological variability does not induce the problems we have raised in this article.

## 4 DISCUSSION

We have outlined why *PC*_*T*_ misleads interpretations when contrasting different temporal snapshots. Further, we have proposed the TPC, which allows for across time points comparisons without impeding the interpretation. Finally, we have also shown that these methods diverge in how much nodal time series correlate and which nodes will be considered hubs. The extent of the divergence between *PC*_*T*_, *PC*_*S*_, and *TPC* will depend on how much the communities fluctuate, the community detection algorithm, the parameters used in the community detection, and time‐varying connectivity method. However, when changing both the time‐varying connectivity method and community detection algorithms, *PC*_*S*_ and *TPC* were consistently the closest to each other, illustrating that *PC*_*T*_ diverges the most.

Measures of network neuroscience aim to increase our understanding about the organization of the brain. Our results show that *PC*_*T*_ can lead to misleading interpretations about what the brain is doing, especially when inferring a property such as integration. At the regional level, the time series between *PC*_*S*_ and *PC*_*T*_ correlated around 0.3 on average during resting‐state (i.e., only sharing 9% of the variance). Our discussion has shown that it is unclear whether changes in *PC*_*T*_ occur due to communities splitting or via increased integration. Hence, interpretations of *PC*_*T*_ being a measure of integration when comparing multiple time points are, in our opinion, ill‐advised. However, averaging over time points is possible for *PC*_*T*_, which multiple studies have done (e.g., in Shine et al. (2016)). This strategy is possible because it is no longer comparing time points with different communities which is unproblematic (but looses the temporal resolution of the PC). In sum, we feel that any quantification of fluctuations of participation through time should use *PC*_*S*_ or *TPC*.

We are not challenging *PC*_*T*_ in all use cases; it is mathematically sound when applied to each time point. The problem arises when contrasting values from different time points that are derived on different community vectors. If two snapshots are contrasted with *PC*_*T*_ and presented with their respective community differences, then each estimate can be useful for understanding each snapshot (e.g., Figure 1d) which can facilitate understanding about the network. However, multiple *PC*_*T*_ estimates cannot be directly contrasted unless you alter the meaning of integration.

The solution we present, *TPC*, does not fit all possible use cases. One limitation is that it can only be applied when the network can return to previous states (the recurrence assumption). Some temporal communities may only be possible after certain events have transpired—for example, during a contagious outbreak, patients (nodes) could form temporal communities in the hospital. Using our proposed *TPC* on such a dataset would entail that postinfection communities influence preinfection participation estimates, which would be unrealistic. Furthermore, care would also be needed for any of the participation methods if a network bifurcates its community structure between entirely different states that have little or no topographic overlap. Thus, the proposed solution only covers networks which can theoretically return to similar states. This assumption appears reasonable for networks such as the brain. However, quantifying variations in how nodes relate to their community assignments (e.g., Bassett et al., 2011) or using time‐varying measures with static communities (e.g., *PC*_*S*_) may be more prudent analysis alternatives. The ultimate lesson here is that network measures need to be chosen based on the knowledge about the system under investigation and the new information that the measures hope to attain.

Here, the focus has been on temporal communities and its recent application within network neuroscience. However, this can also be a more general warning for such nodal measures that are relative to the community structure when applied in multilayer cases. For example, if calculating PC per task when each task has its own community partition will suffer the same interpretability problems. We hope that this article highlights the problematic nature of quantifying temporal nodal measures relative to a fluctuating temporal community partition. We have offered one possible solution for this problem that utilizes temporal community information that does not suffer from similar issues regarding interpretation.

## CONFLICT OF INTEREST

The authors declare no potential conflict of interest.

## Supporting information


**Supplementary Figure S1** Same as Figure 4CD in the main text but showing the relationship with PC_T_ and TPC with PC_S_. A and C show example subjects. B and D show for all subjects.

**Supplementary Figure S2** Replicating the results in Figure 5 using the multiplication of the temporal derivative (MTD) time‐varying connectivity method and TCTC community detection method. (A‐C) The histograms of the correlations of the time series for each method (histogram showing all nodes, sessions and subjects) when using the MTD method for time‐varying connectivity estimates. Everything else in the main text was the same. (D‐E) The histograms of the correlations of the time series for each method (histogram showing all nodes, sessions and subjects) when using the TCTC temporal community detection method for TPC and PC_T_ (PC_S_ is the same as the main text). All other methodological steps are the same as the main text.

## Data Availability

We used data from the Midnight Scan Club resting‐state fMRI (Gordon et al., 2017) that is publicly available on http://openneuro.org (ds000224). The data are available after both preprocessing and denoising steps have been performed (see Gordon et al., 2017 for details of these steps). The data consist of 10 subjects that underwent 10 resting‐state fMRI runs. One subject was excluded due to substantial artifacts. We extracted time series from 200 functionally defined parcels (Schaefer et al., 2018).
