# Supplementary figures and images for: Time‐varying nodal measures with temporal community structure: A cautionary note to avoid misinterpretation
Source: Hum Brain Mapp. 2020 Feb 14;41(9):2347–56. doi: 10.1002/hbm.24950 (PMC7268033; doi:10.1002/hbm.24950)

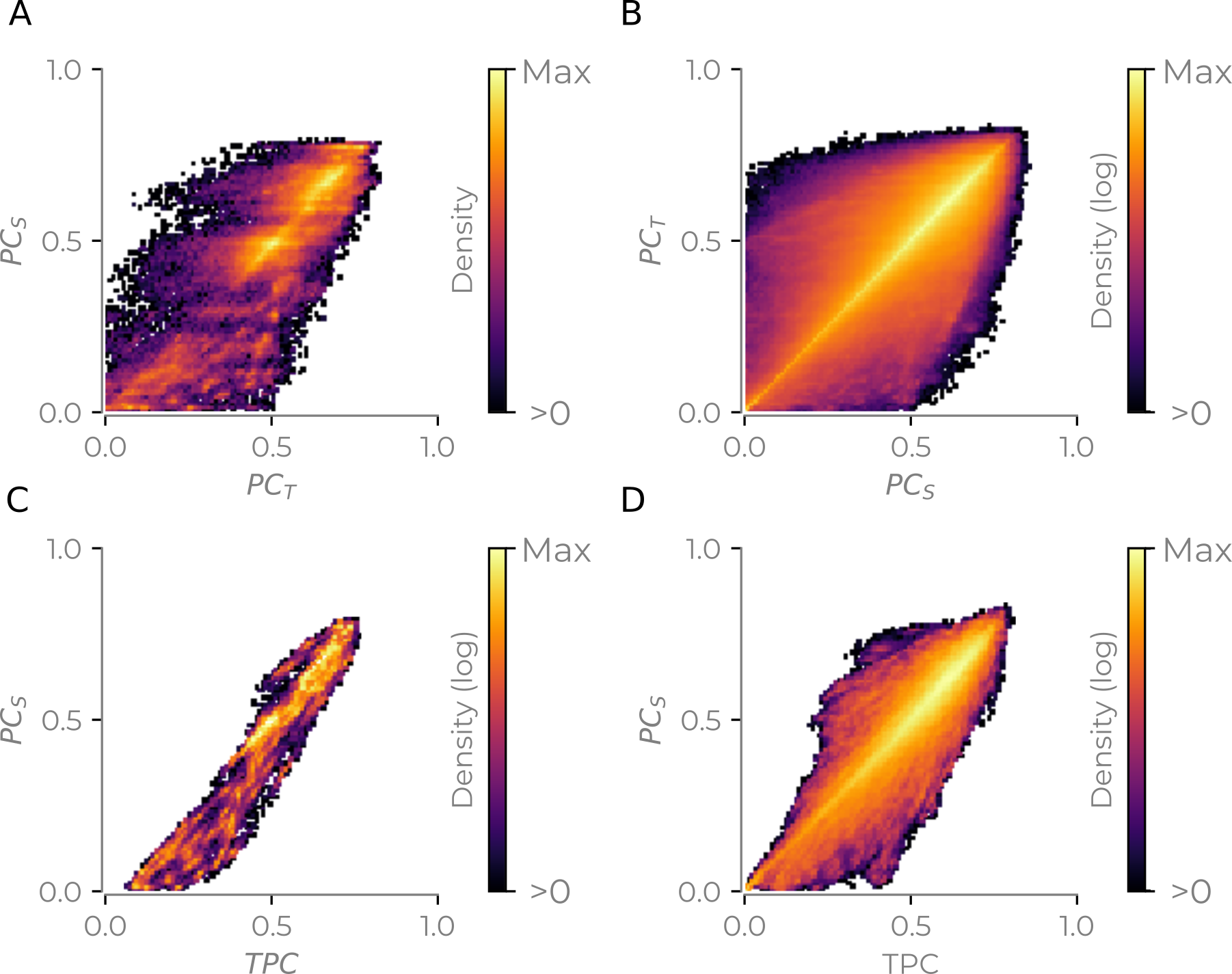

Supplement: Supplementary file 1 — Supplementary Figure S1 Same as Figure 4CD in the main text but showing the relationship with PCT and TPC with PCS. A and C show example subjects. B and D show for all subjects. [file HBM-41-2347-s001.tif]
